# A Serious Game for Patients With Eating Disorders (Maze Out): Pilot User Experience and Acceptance Study

**DOI:** 10.2196/40594

**Published:** 2023-01-27

**Authors:** Maria Mercedes Guala, Kim Bul, Finn Skårderud, Anette Søgaard Nielsen

**Affiliations:** 1 Psychiatric Research Unit Institute of Clinical Research University of Southern Denmark Odense Denmark; 2 Coventry University Institute for Health and Wellbeing Centre for Intelligent Healthcare Coventry United Kingdom; 3 Institute for Eating Disorders Oslo Norway

**Keywords:** eating disorders, serious games, mHealth, coproduction, mobile health, mobile phone

## Abstract

**Background:**

Eating disorders (EDs) are severe mental disorders associated with notable impairments in the quality of life. Despite the severity of the disorders and extensive research in the field, effective treatment for EDs is lacking. Digital interventions are gaining an evidence-based position in mental health, providing new perspectives in psychiatric treatment. Maze Out is a serious game coproduced by patients and therapists that focuses on supporting patients with EDs.

**Objective:**

The aim of this study was to investigate the experiences of engaging in and acceptability of Maze Out among patients with EDs and therapists.

**Methods:**

This study is a qualitative pilot study involving data collected through focus groups and individual interviews and user analytics collected through the game. The participants were recruited from the Odense Mental Health Service of the Region of Southern Denmark. Qualitative interviews analyzed by thematical analysis and interpreted by interpretative phenomenological analysis were used to evaluate the acceptance and experience of Maze Out among patients and therapists. The mobile health evidence reporting and assessment checklist was used to describe the content, context, and technical features of the game in a standardized manner for mobile health apps.

**Results:**

The participants found Maze Out to be engaging, easy to use, and a good platform for reflecting on their disorder. They primarily used Maze Out as a conversational tool with their close relationships, giving them insights into the experiences and daily life struggles of someone with EDs.

**Conclusions:**

Maze Out seems to be a promising tool supplementing the current ED treatment. Further research should focus on evaluating the effectiveness of the game and its potential to support patients with different types of EDs.

## Introduction

### Background

Eating disorders (EDs)—anorexia nervosa (AN), bulimia nervosa (BN), and binge ED—are severe mental disorders characterized by symptoms with behavioral features such as purging, bingeing, and restrictive food intake and cognitive-affective traits such as feelings of fatness and fear of weight gain. A systematic review reported the weighted population mean of lifetime prevalence of AN as 1.4% among women and 0.2% among men, BN as 1.9% among women and 0.6% among men, and binge ED as 2.8% among women and 1% among men [1]. However, the true community incidence of EDs remains unknown.

EDs are complex disorders associated with notable impairments in the quality of life, affecting personal and social life [2-4]. They are characterized by a high risk of comorbidities, such as anxiety disorders (>50%), mood disorders (>40%), and self-harm behaviors (>20%) [5].

Despite the severity of the disorders and extensive research in the field, effective treatment for EDs is lacking. The current recommended treatment worldwide is a combination of nutritional treatment with different forms of supportive therapy or psychotherapy [6-9]. Up to half of the patients with EDs never fully recover after treatment owing to the severity of the condition and frequent relapses. Therefore, there is an urgent need for improved treatment outcomes across the field of EDs [10,11].

The biggest challenges for health care professionals in the treatment of EDs are patients’ lack of insight into their disorder, ambivalence toward recovery, high dropout rates, and weak working alliances between patients and therapists [12]. The ego-syntonic aspects of EDs can partly explain the ambivalence toward recovery and lack of insight into one’s own disorder. Neither individuals who are diagnosed with an ED nor those who are living with EDs but are undiagnosed identify themselves as having a mental illness [13,14]. Dropout rates are high—51% in inpatient treatment and 73% in outpatient treatment among patients with AN and up to 55% among patients with BN [15] (primarily from inpatient treatment). Furthermore, people with EDs face substantial barriers to treatment, such as those related to stigma, access, and affordability, depending on the health care system and governmental support [16].

People with ED also show great variation in the clinical presentation of symptoms depending on both the stage and severity of the disease and the patient’s life circumstances. This among other things implies not only the need for evidence-based treatment in general but also a need for a personalized treatment approach in particular [17].

Overall, the complexity of EDs and the diversity of treatment preferences call for a range of well-designed tools that health care professionals can choose from to meet patients’ needs and improve outcomes [11].

Tools that involve play may contribute to finding new perspectives and insights that cannot be reached in a therapeutic session, particularly when the working alliance is weak. Play provides a context for learning in a fun and engaging manner. Games appeal to aspects that render learning effective and stimulate association among and use of multiple senses [18]. Therefore, serious games (SGs) are of interest from a clinical perspective. SGs are defined as “digital games and simulating tools that are created for nonentertainment use, but with the primary purpose to improve skills and performance of play-learners through training and instruction” [19]. SGs have gained acknowledgment in the fields of health and mental health treatment [20-24], probably because of factors such as play, which is inherently fun. It also allows one to look at a troublesome matter with curiosity while trying new paths without too much risk in real life.

### Prior Work

Digital technologies are considered to enable new insights into the lived experiences of mental disorders. Involving digital technologies may thus enable current treatments to be refined and personalized as well as the generation of new targets for future treatment development [25].

SGs using digital technology have been used in mental health care in various formats. A systematic scoping review by Ferrari et al [26] revealed that so far, most studies on digital game interventions within the context of mental health focused on *youths* aged ≤19 years and that only one study focused on ED (specifically on BN).

Virtual reality (VR) is a digital technology that has been shown to be acceptable as a therapeutic tool for patients with EDs [27,28]. A systematic review of the literature on the use of VR in EDs conducted by Clus et al [28] showed that VR is an acceptable and promising therapeutic tool for patients with EDs. To the best of our knowledge, none of these studies used patients’ active involvement in the production of the game.

Mental health mobile apps also seem to have the potential to expand access to information and support, especially among people who are unable to access face-to-face care. The role of these apps became especially salient during the COVID-19 crisis [26,29].

Although apps are a relatively recent invention in the field of health, there are a huge number of them [30,31]; however, the effectiveness and potential usability of mental health mobile apps for ED are poorly studied [32,33]. The most studied app is a self-monitoring app, Recovery Record [34,35], and the study results suggest that such an intervention is highly accepted by patients with AN and that it could support symptom stabilization or continued improvement as an add-on therapy to usual treatment, if used according to the guidance of therapists [33,35]. To the best of our knowledge, no smartphone app for EDs involving SGs has been developed, tested, or evaluated for its effectiveness, alone or in addition to usual treatment.

Although playing games is a typical activity among children, it is also an expression of an inner drive and makes it possible to break out of habitual behavioral patterns and try something different [36]. Social scientists have identified capacities generated during playing, including the ability to find meaning in experiences [37], metacommunication [38], affect regulation [39], and self-transformation [40,41].

Playing also has a pretend aspect, which can allow players to prepare for something they both want and fear—to get well. According to Winnicott, a well-known English pediatrician and psychoanalyst, it is by playing that an individual can be creative and use their whole personality, thus discovering themselves [42].

In the landscape that Winnicott draws, the actual toy is manufactured within the playful process; that is, although the child is given toys, only they can activate them and make them work as such. Winnicott postulates that it is creative apperception more than anything else that makes an individual feel that life is worth living [42]. This encourages the production of SGs in which players can shape the game and thus activate their creative perception.

### Coproducing Technologies

The active involvement of patients and clinicians in the development of technologies is central to their success as a part of treatment [25]. It is also a path toward increasing the feasibility and appropriateness of technologies for use by patients because they should fit patients’ needs and goals [16,17]. Adequate training and support for users are important for the successful implementation of technologies [25].

An SG named Maze Out was developed at the Psychiatric Hospital in the Region of Southern Denmark from January to December 2020 by a close collaboration among 4 patients with EDs, 3 therapists, and a commercial game company. The game was intended to supplement usual treatment. The development of the game was driven by the concept of coproduction. Coproduction is “a form of partnership working and an approach to service development and practice which brings together those who use services and those who provide them” [43]. The coproduction process included six 4-hour face-to-face workshops, regular mail correspondence, and a private chat forum to discuss small amendments after each iteration of the game.

The tasks that the coproduction team had to work on were (1) defining the mechanics and style of the game, (2) describing situations that challenge people with ED, and (3) creating reflection exercises based on those situations.

The first workshop included an introduction and a mutual explanation of what it meant to be part of the coproduction process. It was emphasized that all the participants should be willing to provide support by creating new knowledge and focusing on the importance of sharing different perspectives. It was explained that power would be shared equally among all the participants, based on the recognition of their different areas of expertise.

The second workshop focused on the mechanics and look and feel of the game. The focus was on creating a flow experience. The approach was iterative. The idea was to give the player an experience of progression with an end goal: to come out of the maze. The journey was divided into days. Each day had a beginning, challenges, and an end with a reflection exercise. The look and feel of the game was initiated by a film producer who had an ED and was collectively defined by the whole team.

The subsequent 5 workshops focused on the creation of challenging scenarios and situations. The objective was to make them playful and at the same time meaningful, thereby providing a balance between humor and the seriousness of having an ED. The therapists presented reflection exercises. All the contents were shared within the team, and final decisions were made jointly during the workshops, examining what was more feasible and relevant.

The last workshop focused on finalizing the first prototype. Focus groups and individual interviews were used for process evaluation.

### Mobile Mental Health Game: Maze Out

Coproduction sessions resulted in the first prototype of the game “Maze Out.” Maze Out is a digital SG to be played on an Android tablet or smartphone and constructed as a labyrinth, a maze. The goal of the game is to get out of the maze and establish the desired life. The desired life is not presented to the player as something concrete and specific to the game but is rather a metaphor aimed at inviting the player to get in touch with their own dreams and desires.

**Figure 1:**
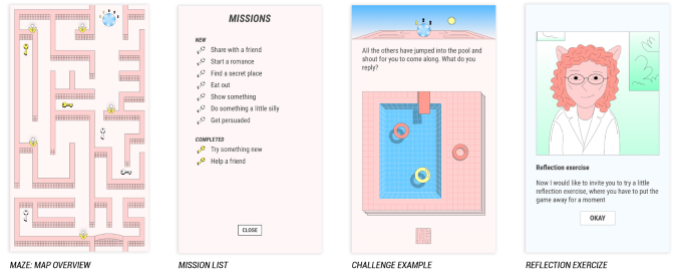
Overview of the design, missions, and aesthetics of Maze Out.

When playing the game, the players are presented with various scenarios and choices that represent everyday life. They must complete 10 missions (Figure 1) through the maze to find their way out. The missions reflect different themes embedded in the game, including not only challenges related to food and exercise but also those related to feelings, relationships, and communication.

When playing Maze Out, the players are asked to perform reflection exercises and deal with the challenges on their journey, with the purpose of helping them relate the game content to their everyday lives. The challenges change as players progress toward psychologically more demanding situations, becoming more metaphorical and placing an emphasis on complex psychological processes, such as setting limits, making decisions autonomously, and managing emotions.

To that best of our knowledge, Maze Out is the first SG coproduced and put into practice by therapists and patients in the field of EDs. Therapists contributed by participating in the preparation of the focus group and interview guides used for the evaluation. Patients contributed as buddies to the participants, offering advice and support on how or when to use Maze Out.

### Goal of This Study

As the first step in assessing Maze Out as an important tool for ED treatment, this study investigated whether the game is usable, acceptable, and meaningful among patients and therapists.

The aim of this study was to investigate the experiences of engaging in and acceptability of Maze Out among patients with EDs and therapists.

## Methods

This is a qualitative study involving data collected data through focus groups and individual interviews and an analysis of small amount of user analytics collected through the game. Patients with EDs and a therapist, who had not been involved in the development process of Maze Out, were invited to participate in focus or individual interviews and evaluate Maze Out. The therapist was interviewed separately from the patients.

### Recruitment

A total of 20 female patients with a mean age of 24 (SD 5.29; range 21-45) years who were diagnosed with AN, BN, other EDs, or EDs unspecified (International Classification of Diseases 10th Revision codes: F.50.0-F.50.9) were recruited. They received treatment from the Odense Mental Health Service in the Region of Southern Denmark, were informed about this study, and provided written informed consent. During the study, 10% (2/20) of participants dropped out because of demanding life circumstances. A therapist from the Odense Mental Health Service in the Region of Southern Denmark, who did not participate in the coproduction activities, also participated in this study with the same premise as the patients (Textbox 1).

**Inclusion and exclusion criteria.**

- Patients’ characteristics
  - Inclusion criteria:
    - International Classification of Diseases 10th Revision (ICD 10)—diagnosis of eating disorders (EDs): F.50.0-F.50.9 (ICD-10 codes for EDs)
    - Age: 20 to 70 years
    - Treatment: receiving treatment at the Eating Disorders Unit of the Odense Mental Health Service in the Region of Southern Denmark
    - Interest in the game: yes
  - Exclusion criteria:
    - ICD 10—diagnosis of EDs: F.20.0-F.20.9 (ICD-10 codes for schizophrenia, schizotypal, and delusional disorders)
    - Age: <20 years
    - Treatment: not receiving treatment
    - Interest in the game: no
    - Participated in the coproduction of Maze Out
- Therapist’s characteristics
  - Inclusion criteria:
    - Interested in trying the game
  - Exclusion criteria:
    - Participated in the coproduction of Maze Out.

### Study Procedure

#### Overview

**Figure 2:**
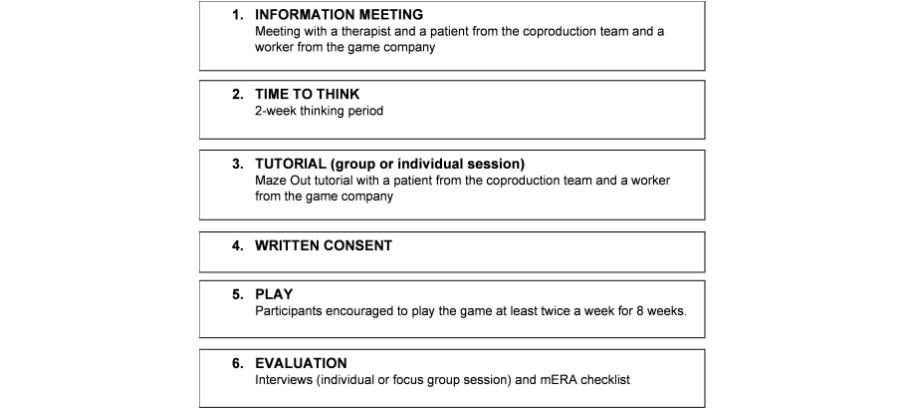
Study flow from recruitment to evaluation.

The study flow of participants is presented in Figure 2. Each participant attended an information session in which they were informed about the study procedures and provided the opportunity to ask questions. The patients had 2 weeks to consider their willingness to participate in the study. Those who were willing to participate were instructed to download the game on their mobile phones and were instructed to play it at least twice a week within an 8-week period. The game was presented to the participants in a 1.5-hour group or individual session. The sessions were facilitated by 2 members of the coproduction team, wherein the participants were encouraged to try Maze Out and ask questions about it. A patient and a therapist from the coproduction team made a video for the participants that presented information about the game.

The participants were also contacted by the study team to sign an informed written consent form. They chose a fictive player name that was only known by themselves and the therapist in charge of the study and a personal password, which they did not share with the therapist.

During the study period, the participants could choose to be contacted by a buddy for support if they had questions about the game and for them to be reminded to play the game. The buddies were the patients who participated in the coproduction of Maze Out, and they signed a confidentiality agreement before the start of the study. After the 8-week period, all the patients and the therapist were invited to an in-depth interview, and they could choose between participating in an individual interview or in a focus group session.

#### Data Collection

The quantitative data collected in this study consisted of user analytics. Qualitative data were collected through focus groups and individual interviews, which were performed using semistructured interview guides to ensure that all relevant information was brought to light and the data were saturated (Multimedia Appendix 1). All the interviews were audio recorded and transcribed verbatim, with the consent of the participants. In total, 60% (12/20) of patients participated in focus group sessions—3 sessions with 4, 5, and 3 participants, respectively. Individual interviews were help with the therapist and 20% (2/20) of patients.

#### Data Analysis

We used the mobile health evidence reporting and assessment (mERA) checklist (Multimedia Appendix 1) to describe the usability of the game. The mERA checklist is a 16-item checklist that aims to standardize reporting and enables the quick assessment of eHealth and mobile health apps [44].

To explore to what extent, how, and why the participants found playing Maze Out meaningful, interpretative phenomenological analysis (IPA) was used to interpret the interview data [45]. IPA is a phenomenological approach used to explore how people make sense of their life experiences. IPA also involves a hermeneutic element that is used in the interpretation of the experiences. The researcher needs to interpret the accounts given by the participants to understand their experiences. IPA involves navigating through different layers of interpretation as one engages deeply with the texts of participants’ personal experiences [46]. A qualitative analysis of the interviews with the patients and therapist was conducted, and the mERA checklist was used as a structuring tool for reporting the findings. Thus, the analysis to some extent also draws upon the principles of a thematic analysis approach when it comes to presenting the findings [47]. Thematic analysis is a widely used method for identifying and describing themes both emerging from data and being meaningful relative to the research question [47].

#### Measures

The mERA checklist briefly addresses the technology’s infrastructure, platform, interoperability, delivery, content, testing, access, costs, adaptation inputs, limitations, replicability, and data security [44].

The use of Maze Out during the study was summarized using descriptive statistics on the average game duration and frequency of use.

The group and individual interviews were conducted and analyzed by the first author (MMG) and a trained research assistant. The interviews were audio recorded, notes were taken, and data were analyzed without any computer-assisted qualitative software. All the recordings were transcribed. The analysis was primarily carried out by a dialogue between MMG and the research assistant to gain a deep understanding of the phenomena and was sent back and forth to the fourth author, ASN, who evaluated and gave feedback on the analysis. ASN also had full transcriptions and focused on what might have been missed or required further exploration. The patients’ experiences with the game were analyzed in relation to whether they found the game useful and how it supported them. The patients’ opinions about the game as an add-on to the usual treatment were interpreted, and the reactions they would have had if they had shared the game with their close relationships were explored.

### Ethics Approval

The local ethics committee from the Region of Southern Denmark was approached, but because the study was noninvasive and only investigated the patients’ perception of a SG offered as an addition to usual treatment, it did not require approval from the committee (reference number 20202000-66). All the participants provided informed consent before their inclusion in the study. All guidelines for data protection were followed. All study procedures were performed in accordance with the second Declaration of Helsinki and Good Clinical Practice guidelines. The study was registered by the Danish Data Protection Agency (reference number 20/19414).

## Results

### Overview

The main description and first brief evaluation of Maze Out according to the mERA checklist are presented in Textbox 2.

**Mobile health evidence reporting and assessment checklist.**

1. Infrastructure: the game requires a phone or tablet no older than 5 years and running Android (Google LLC) or iOS (Apple Inc).
2. Technology platform: Google Firebase (Google LLC) was the technology platform used. Maze Out is a single page app. Backend is coded in NodeJS. Express server was used to handle calls to backend. Frontend is coded in ReactJS.
3. Interoperability: the game was not directly connected to the health organization system. The player can send an email to themselves with the content of and reflections from the game and then bring them to therapy.
4. Intervention delivery: all the patients were encouraged to play the game twice a week. The patients were offered to be contacted by a patient from the coproduction team during the project.
5. Intervention content: the game was developed in close cooperation with 4 patients, 3 therapists, and 3 workers from a game company.
6. Content testing: all the patients thought that Maze Out could be used as an add-on tool to their current treatment. All the patients thought that the game should be played outside therapy. Some patients suggested that the game could be used in group therapy or with relatives.
7. User feedback: all the patients found Maze Out to be easy to use. Feedback collected from the patients during focus groups and interviews has been described in this paper under the *Results* section.
8. Access of individual participants: all the patients thought that the game could be used for all eating disorders. Some patients asked for more personalized versions of the game.
9. Cost assessment: Maze Out can be downloaded free of charge. The domain name, hosting, and use cost about US $800 per year per 1000 patients.
10. Adoption inputs: a 1.5-hour group or individual session was conducted by 1 patient from the coproduction team and 1 person from the game company.
11. Limitations for delivery at scale: it will not be possible to offer support to the players from the coproduction team.
12. Contextual adaptability: the prototype has been translated to English but not tested yet. Suggestions for tailoring and further modification has been described in the *Conclusions* section.
13. Replicability: if the content is adapted, Maze Out has the potential to be used for other mental disorders.
14. Data security: usernames are anonymous. All passwords are stored in encrypted form.
15. Compliance with national guidelines or regulatory statutes: all the patients were receiving usual treatment at a mental health department.
16. Fidelity of the intervention: the intervention was delivered as planned. Participant engagement has been described in depth in this paper.

### User Analytics

The participants reported that what worked best for them was playing when they felt like it. Back-end data showed a wide variation in the number of times the participants played the game (5 to 38 times) as well as in the duration of playing (a few minutes to one and a half hours per session). The number of completed missions ranged from 4 to 21 (when completed, the missions could be played again).

### Participants’ Experiences and Views

#### Theme 1: Staying Motivated and Engaged

All the participants agreed that the situations and scenarios presented in the game were relatable to those in their own lives and were relevant and important to them. The game’s missions represented different everyday situations for them, and this encouraged reflection on their own situation and kept them engaged:

Overall, I actually think it’s a really, really nice game. I feel like I can recognize myself in really many of the situations and see myself in it. Then I thought it made good sense. I thought it reflected a lot of the things, the dilemmas and the thoughts you have during your disease.

P07

...it can also make me really sad how many things I just suddenly discover that I actually have problems with. Things that I may not have thought about have something to do with the eating disorder, but things you choose from...because you need things to be very structured around your eating.

P01

...such as when you are on the beach and meet your friends and they ask if you want to join and such things—there are some of the challenges that I can well recognize, and where I think they make you think...

P06

Several participants described that they felt relieved when presented with a situation that was a direct representation of their own lives:

...that there are many of the things where you think ‘uh, how cool is it to be asked about this, because I do too, and then it’s not completely abnormal then.

P13

It’s really nice for me to see and hear that some of this silly thing I do with my shopping cart, there are apparently others who do since it’s in the game.

P01

Most participants expressed that the game’s reflection exercises were a crucial factor in deciding whether they continued to play. They found the reflection exercises challenging and rewarding, as they felt successful when completing them. The participants expressed that the missions supported a good game “flow” and that it was motivating when a mission was completed:

I thought it was really cool to have those keys and missions. There was such a good continuation in the game.

P08

...and when I am then rewarded and actually get a key to it—there I become like that completely “arh” (satisfying-sigh). I thought that was very nice.

P10

#### Theme 2: the Game as a “Vehicle” to Help Significant Others Better Understand What Living With ED Means

Several participants felt that the game was a good tool for communicating with their relatives about their disorder and symptoms. They experienced that their close relationships gained awareness and understanding of ED from talking about the game:

It makes good sense to let our parents play the game so that they can also see what dilemmas one can face...Because I find that it can be difficult to put into words or describe how one feels because it is sometimes a bit abstract, the way we think on...So at least I thought it was a really good tool to show one’s relatives, because then they might get a little better acquainted with it.

P07

...the person in my life who has the hardest time understanding my eating disorder, that’s my dad. There I have actually been able to use it, I could kind of talk to my dad about it and tell him that this [event in the game] is what happened while we were sitting and eating together where I had to get off the table.

P09

There was strong agreement among participants about the game being a more appealing tool to relatives than the existing analog. Their experience was that Maze Out could be used to initiate a dialogue about ED with close relationships. One of the participants described that she had difficulties in initiating a conversation about her disorder with her mother and boyfriend, and the mother and boyfriend found it very overwhelming to participate in an information meeting with relatives. Maze Out provided the relatives with information regarding how EDs work in an uncomplicated manner. Another participant mentioned that it was easier for the relative to play the game than to contact services that offer advice to relatives:

It is definitely more appealing that it is a game rather than for example a book or folder of papers. I have for example thought about my one little sister, she does not understand it, and that is fair enough, because she does not run around with it. And she can easily page and search the web for the different things, but it would just be more intuitive with the game.

P10

#### Theme 3: the Content in Maze Out and Therapeutic Considerations

The participants liked the content of Maze Out and found it adequate and relevant.

Many participants felt that the missions were repetitive. Some found this experience frustrating and a reason to discontinue playing. Other participants’ attitude toward the repetitions was different, as it dawned on them that the missions reflected their own lives:

On the one hand, it makes sense that it [the same result] comes again, because it does in real life, when one keeps making the same choice.

P10

From the perspective of the participating therapist, repetition was a valuable tool because it could show the patients that when they experience frustration over ending up in the same place, a resort may be to choose differently:

It is appropriate in relation to help patients to make different choices. You could also say that patients keep coming back to the same situations in their lives.

B01

The participants experienced acceptance throughout the game in situations where they may tend to blame themselves:

Sometimes you get the feedback: “that it was probably also the best in these situations,” and I thought so...because you spend so much energy to knock yourself in the head and say “why can’t you let it go? Why can you not just be normal like everyone else?” So, it is good to understand that, that you’re good enough anyway...

P14

The participating therapist expressed enthusiasm regarding the validation the game provided by recognizing the choices that the patients usually make. Here, validation was described as appropriate for processing the patients’ self-blame, which is the same as the what the patients themselves mentioned. From the therapist’s perspective, it was also assessed whether the game gives patients the courage to choose actions other than those that they would normally choose.

#### Theme 4: Maze Out as a Valuable Add-on to Treatment as Usual

The participants were positive about the game being an addition to their current treatment:

Well, where I heard what I thought “yes, it’s exciting. This is something new.” And because it’s a game and it’s a bit like Tinder [a dating app], it’s something we all know. So maybe that makes it not so sad and boring to be in treatment.

P14

When asked specifically about what Maze Out might add to the existing treatment options, both participants and therapists highlighted the opportunity to try out new actions without risk and its accessibility in everyday life:

So, I actually thought that in the periods where I’ve struggled, it’s been great to be able to come in and play a lot. So, I could easily play a little every day. So instead of scrolling through Instagram, sit back and play, and try to make some good decisions and get a little better.

P13

The participants described that the game gave them the opportunity to work on their disorder in everyday life, which the therapeutic space does not always reach:

If you take my situation where I’ve been to T [therapist] for interviews, and then I come home and have kind of put on the other mask. And the game has been good to use. I can kind of work with the eating disorder through the game because I get some reflections when I’ve just talked to a friend or my boyfriend or family.

P14

Several of the participants also mentioned that the game would be suitable for direct use in group therapy:

If you were in a group and you all had this game, so that you might be able to take some topics up that road. Because sometimes it can be difficult to figure out what to say and such. So, if you then had some specific topic, then you could be inspired by each other. I also think it could be used for that. It gives such a feeling that you are not sitting alone with these thoughts that you can now have in your head.

P05

Several participants also highlighted that the game would be a fun and an easily accessible addition to the treatment that might otherwise feel harsh and sad. One of the participants felt that she would have benefited from it during treatment, as it would have allowed her to work with herself in a more comfortable manner than the existing treatment options. Most participants valued the possibility of being able to use the game exactly when they felt motivated or ready to work with themselves and their ED, and not just when they were in therapy. However, some participants felt that it was important to have some self-insight before the start of the game, as otherwise, there would be too much denial about the situations and not enough reflection. One of the participants expressed this when talking about the beginning of her treatment by saying that she “actually did not think it was anything wrong.”

#### Theme 5: Timing of the Intervention

Most participants considered that it would be appropriate to introduce Maze Out at the start of their treatment because it might be “an eye opener.” One of the patients stated the following:

It would make sense to play at the beginning of starting a course, because I think it can open your eyes to how many places in your life, it inhibits like that or that it is an eating disorder thought you have.

P06

The participants added that through the game, they were able to see how they had developed in several areas during their treatment:

I would definitely also say that it will make sense to start with. This way you can see for yourself where you have some challenges or problems. But I also thought it could be a good tool at the end of one’s course of treatment, to see how far you have moved or developed. Because I have experienced that I sometimes have doubts about how much I have moved and which areas I have moved in.

P07

Another participant agreed and said the following:

It gave me such a good feeling because I could well see that two years ago, I had done it here and now I do not want to do it. So it also gave me such a bit...in the form that it was dilemmas, which at one time had been much bigger dilemmas.

P08

#### Theme 6: Future Recommendations for the Game

##### Individual Adaptation of the Game

After trying out the game for 8 weeks, several participants proposed the idea of individual adaptation of the game, that is, adapting the game according to each user’s diagnosis and associated needs:

I have been thinking about, whether it would make good sense if you (the game) had a bit more in-depth information about the person who was going to play it beforehand, and you (the game) then found out in that way whether it was anorexia, bulimia, or BED, or what is it that you struggle with and then adapted it that way. Yes...

A01

##### More Content

Most participants recommended developing more content so that they could continue playing with new scenarios:

Umm...in any case, I think there should be a few more situations, so that even if you have got a key and completed it, you may well end up back in the same scenario again. And there I thought it would be annoying that even if you answered something else, you would come back to the same point. And it could help—I know it’s challenging to program—to put in a few more situations.

C01

##### Reminder

The participants also found it important to add an automated reminder to play that would appear as a push notification on their mobile device.

Several participants mentioned that they would benefit from notifications because they experienced forgetting the game if they were in a “bad period” and that it was precisely during this period that they would benefit from playing the game:

Because if I know myself, I believe that I will refrain from getting it played if I am in a bad period. Then I think I wouldn’t want to relate to things [ED symptoms], but then it was probably what one had to do. So it would be really nice to have some reminders so that you get it done.

L01

Some participants were annoyed about the repetition of some situations during the game, which had consequences on their desire to play:

Well, I thought it was...I got actually annoyed with it eventually and stopped playing. Because it was so annoying that it was the same questions that came. Where it was a bit like “well, I’ve answered that.” I just didn’t feel like I was progressing. And it was also the case that for some of the questions I did not want to answer this or that. I didn’t want to answer any of that. and it’s just like why is there a question about throwing up when that’s not what I’m struggling with. It was such that I could not use it for anything...Oh, I’ve spelled it out...

A01

## Discussion

### Principal Findings

Maze Out seems to offer a safe space that allows players to reflect upon the challenges associated with EDs. The way in which the content is presented in the game, including the playful aspect, plays a crucial role in enabling users to relate the content of the game to their experiences with EDs.

In this study, the participants emphasized the importance of the game representing situations that were a direct representation of those in their own lives; they felt relieved, and it helped them stay engaged. This aspect of the game is probably a result of the coproduction process in which patients were asked to provide inputs on the content of the scenarios.

During the study period, the participants could choose to be contacted by a buddy for support if they had questions about the game and to be reminded to play. All the participants, except for 2 (10%), chose to be supported by a buddy via a closed chat forum. They were all active on the closed chat forum and found it useful in terms of being encouraged to play. This support from buddies was meaningful for the participants but is difficult to be sustained in the future. A possible way to resolve this could be to organize this task to be taken over by new or existing peer support workers within the mental health service, in which case, the peers should be trained in how to introduce and support the use of Maze Out. Members of the coproduction team might deliver such training, which might make it possible to implement Maze Out in routine care. Creating tutorials aimed at new patients and new staff might be another option to help implement Maze Out. A “Train-The-Trainer” model can be very cost-effective and successful in creating capacity while rolling out Maze Out in clinical practice [48-50].

The participants found Maze Out useful as a vehicle to help close relationships better understand what living with EDs means. The playful aspect of Maze Out stood out in making it an engaging tool for talking about EDs and more useful than the existing offerings.

Talking about mental illness with relatives can be difficult, especially when the person is ashamed of it. Maze Out probably facilitates overcoming this because the game appeals to young people (in the case of siblings and peers) and is easily available. The fact that Maze Out is based on everyday situations enables patients to use the game as an immediate tool to be applied in “real life” and enables contact with the inner material, which is not always possible in therapeutic sessions. The game’s mobility and accessibility give the possibility to play it in a safe environment and at a time when the participants thought reflection is most required and relevant.

The participants experienced repetitions in the game as annoying, whereas the therapist found them valuable because they would probably encourage patients to choose differently.

According to the participating therapist, another strength of the game is the ability it provides to experiment with different actions within a safe environment. The participants described this as not having to make decisions based on what they felt was expected of them and feeling supported by Maze Out regardless of the choice made. By trying out different scenarios in the game, they could gain self-confidence in decision-making.

A state of flow in a game can be achieved by carefully balancing 2 extremes: the state of anxiety, which is usually a consequence of the challenge at hand being much greater than a person’s existing skill level, and the state of boredom, which occurs when a person’s skill set is far greater than what is required for the challenge [51]. The results of this study underline the importance of finding a balance between the possibility of making new choices and the boredom or irritation associated with most repetitive actions. A good balance could be achieved by observing the flow state of Maze Out in cooperation with patients, therapists, and game experts.

The participants agreed about the usefulness of Maze Out in ED treatment. However, it is important to keep in mind the possibility that the user avoids therapy, thinking that Maze Out is enough treatment. Although patients could be informed about Maze Out not being a treatment tool by itself, we think this misunderstanding could be possible, as therapy is much more demanding than a game. The participants suggested using Maze Out in the therapy session when they get stuck and using it as a conversation tool. This could be a way to enhance the current treatment.

The results indicate that Maze Out could be used at all stages of treatment. At the beginning of treatment, it could be used to motivate patients to continue their treatment; during the treatment, it could be used as a platform from which to start a conversation with the therapist or members of a therapy group; and at the end of the treatment, it could be used to be aware of the extent to which they have improved during treatment.

Making an individual adaptation of Maze Out, as the participants suggested, would imply missing the possibility for the game to help patients with experienced symptoms that do not fit the current diagnosis or to gain insight when they change symptomatology during their treatment, which often happens in this patient group [52].

Another adaptation to Maze Out to consider is to test it in different psychiatric populations that have an ED as a comorbidity. The prototype has been shown to be useful in the Danish health care context and has been translated into English to see whether it would be helpful in the context of the national health care system.

### Limitations

One of the limitations of Maze Out and thus of the present pilot study is the relatively limited variation in the content in Maze Out. More varied content, according to different ages and life circumstances, would probably increase the usability of the game because more patients will be able to relate to the situations that the game presents.

This limitation is probably because of the limited number of patients who participated in the coproduction phase. They had different ages and different life experiences and were able to contribute to content variety. However, there was a point of saturation regarding the scenarios they came up with. For further development efforts, it is be important to involve new patients to create new game content.

It is relevant to further examine when it is best to offer Maze Out and, more importantly, to whom. The participants indicated that the game would probably help during the onset of EDs or at the initiation of treatment, as Maze Out is engaging and accessible. As most EDs have their onset during puberty, alliance at that stadium is crucial. Maze Out could be a promising tool to prevent increase in severity and promote early intervention. The participants might also benefit from the game when it is used several times during their treatment. It could give patients the opportunity to look at how their choices change during the treatment and how they have changed.

This study has a series of limitations and strengths. The described perspectives and experiences stemmed from a group of participants who were approached and agreed to try the game. Most patients who were invited agreed to try the game, but we did not contact those who declined to explore why they did so, and this may be regarded as a limitation. Likewise, our findings must be considered a snapshot of how Maze Out is experienced.

We cannot conclude whether playing Maze Out has an impact on symptoms, insight, relationship with family, and network or how it relates to the treatment course in the long term. This study only reflects the personal experiences and views of 18 patients and a therapist, who accepted the invitation to play the game, shortly after having played the game. However, this is also a strength of the study. This study describes the usability of and experiences with the game among diverse patients, representing different ED diagnoses and a rather large variation in age. Knowledge of the patients’ views and experiences is crucial for the potential implementation of the game in clinical practice because attractiveness and meaningfulness are central for continued use. It can also be considered a strength of this study that the experiences of the therapists are also included, albeit to a minor extent.

### Conclusions

Maze Out appears to be useful and accepted as an add-on tool to treatment in EDs. The results of this qualitative pilot study indicate that the next step should be to work on another iteration of this first prototype in which more missions with greater variety are incorporated. Future research should focus on evaluating the effectiveness of the game and its potential among patients with different types of EDs.

## Appendix

Multimedia Appendix 1 Interview guide used in the individual and focus interviews.

## Abbreviations

AN: anorexia nervosa
BN: bulimia nervosa
ED: eating disorder
IPA: interpretative phenomenological analysis
mERA: mobile health evidence reporting and assessment
SG: serious game
VR: virtual reality
