# Supplementary material for: A Serious Game for Patients With Eating Disorders (Maze Out): Pilot User Experience and Acceptance Study
Source: JMIR Form Res. 2023 Jan 27;7:e40594. doi: 10.2196/40594 (PMC9919769; doi:10.2196/40594)
Supplement: Multimedia Appendix 1 [file formative_v7i1e40594_app1.docx]

# Interview guide

## Can you describe your experience of the game?

How have you used it?

Can you give an example?

## Have the missions made sense?

Why or how?

Are there any of the missions that have been particularly meaningful to you?

Can you give an example?

1. Have the reflection exercises made sense to you?

Have you been able to use them for anything?

Can you give an example?

## If you have used the game over a longer period, has it made a difference to your experience?

What has it meant?

## Does it matter that it is a game compared to other forms of treatment/approach?

If so, how?

Can you give examples?

1. Have you shown the game to your relatives?

Do you think they could/will be able to get something out of trying out the game?

What do you think they will be able to get out of it?

## Do you think that the game could be used as part of treatment?

Why? How? (if yes) Where in the process?

## Was there anything you missed in the game?

If so, what?

## If we want to improve the game, what would you suggest we change/add/delete?
